# Low opinion of generic oral anticancer drugs: an unacknowledged factor resulting in medication waste in China

**DOI:** 10.1093/oncolo/oyag057

**Published:** 2026-02-23

**Authors:** Yan Wang, Hongwei Song, Jinming Cao, Zhicong Chen, Feng Xu

**Affiliations:** Department of Pharmacy, Sixth People’s Hospital South Campus, Shanghai Jiaotong University, Shanghai 201400, China; Department of Pharmacy, Sixth People’s Hospital South Campus, Shanghai Jiaotong University, Shanghai 201400, China; Department of Pharmacy, Sixth People’s Hospital South Campus, Shanghai Jiaotong University, Shanghai 201400, China; Department of Pharmacy, Sixth People’s Hospital South Campus, Shanghai Jiaotong University, Shanghai 201400, China; Department of Pharmacy, Sixth People’s Hospital South Campus, Shanghai Jiaotong University, Shanghai 201400, China; Department of Pharmacy, Shanghai Deltahealth Hospital, Shanghai 200125, China

Hospital medication waste, comprising both hazardous and nonhazardous products, includes unused or expired drugs in the medicine supply chain.^1^ In this regard, Mackler et al. provided a solution for increasing access to anticancer medication while decreasing medication waste.^2^ Paradoxically, anticancer medication waste and cancer drug shortages often occur simultaneously. Thus, this commentary focuses on oral anticancer medication waste in China.

Since China is a developing country with a large population and vast territory, its medical resources are extremely limited. Despite the country’s significant drug shortage problem, medication waste is a common issue. Consequently, the China National Health Commission called for curbing drug waste in 2023.^3^

As for the cause of medication waste in China, it is multifaceted. From the perspective of oncology pharmacists in ordinary healthcare institutions, there are two possible causes. The first is dosage container size. In oncology pharmacy practice, surplus drugs are occasionally left in single-dose vials to meet individualized dosing requirements, after which the residues are typically discarded. The second cause is the low opinion regarding generic oral anticancer drugs, resulting in medication waste.

Currently, the National Volume-Based Procurement (NVBP) of drugs policy is being implemented in China. As the world’s second-largest pharmaceutical market, the NVBP has achieved significant price cuts, improving bargaining power by pooling purchasing power across the country.^4^ In this case, procurement volumes are guaranteed for bid winners to achieve lower drug prices. Meanwhile, the National Healthcare Security Administration requires that public hospitals fully implement volume-based procurement of drugs in a normalized and institutionalized manner. Under this setting, public hospitals (and most private hospitals) in the country must purchase a quota of NVBP drugs without compromise.

Unfortunately, although the drugs listed under the NVBP have passed the Generic Consistency Evaluation, many Chinese people do not acknowledge them. Regarding anticancer drugs, they consider them as invalid or counterfeit products.^5^^,^^6^ Additionally, since each physician is assigned a quota for NVBP drugs, he/she might prescribe such ultralow-priced drugs to friends and family members but then discard them as medication waste. As for the patients, there is poor medication compliance, with many simply discarding the NVBP drugs when leaving the hospital.

It is important to note that extremely low drug prices do not necessarily indicate low drug quality. However, most Chinese people adhere to the belief that they “get what they pay for” when it comes to medication. In this regard, they believe that the quality and efficacy of NVBP drugs are inferior to those of original or brand-name drugs. Lowering the price of generic drugs was an attempt to provide good health care for the many Chinese citizens but had the unintended effect of making some people feel the drugs would not be valuable. As a compromise, the government should set appropriate drug prices instead of simply lowering them to the level of minimal cost. This action will not only provide pharmaceutical companies with appropriate profit margins but also help them maintain the quality of NVBP drugs, ultimately strengthening public confidence in such products. This may be a key component for resolving the problem of medication waste in China.

## Conflicts of interest

None declared.
